# Synchronously wired infrared antennas for resonant single-quantum-well photodetection up to room temperature

**DOI:** 10.1038/s41467-020-14426-6

**Published:** 2020-01-28

**Authors:** Hideki T. Miyazaki, Takaaki Mano, Takeshi Kasaya, Hirotaka Osato, Kazuhiro Watanabe, Yoshimasa Sugimoto, Takuya Kawazu, Yukinaga Arai, Akitsu Shigetou, Tetsuyuki Ochiai, Yoji Jimba, Hiroshi Miyazaki

**Affiliations:** 1 National Institute for Materials Science, Tsukuba, Ibaraki 305-0047 Japan; 2 Nihon University, Koriyama, Fukushima 963-8642 Japan; 3 Tohoku University, Sendai, Miyagi 980-8579 Japan

**Keywords:** Metamaterials, Nanocavities, Optical physics, Electronics, photonics and device physics

## Abstract

Optical patch antennas sandwiching dielectrics between metal layers have been used as deep subwavelength building blocks of metasurfaces for perfect absorbers and thermal emitters. However, for applications of these metasurfaces for optoelectronic devices, wiring to each electrically isolated antenna is indispensable for biasing and current flow. Here we show that geometrically engineered metallic wires interconnecting the antennas can function to synchronize the optical phases for promoting coherent resonance, not only as electrical conductors. Antennas connected with optimally folded wires are applied to intersubband infrared photodetectors with a single 4-nm-thick quantum well, and a polarization-independent external quantum efficiency as high as 61% (responsivity 3.3 A W^−1^, peak wavelength 6.7 μm) at 78 K, even extending to room temperature, is demonstrated. Applications of synchronously wired antennas are not limited to photodetectors, but are expected to serve as a fundamental architecture of arrayed subwavelength resonators for optoelectronic devices such as emitters and modulators.

## Abstract (web summary)

Applications of metasurfaces for optoelectronic devices require wiring to each isolated antenna for biasing and current flow. Here, the authors report optimal wire interconnects design for controlling the optical properties and present antenna-enhanced mid-infrared photodetection incorporating a single quantum well.

## Introduction

The manipulation of electromagnetic waves with arrayed subwavelength resonators has long been a major topic in electronic engineering. Yagi–Uda antennas generate sharp beams from a half-wave emitter through near-field mutual coupling with parasitic elements arranged with deep subwavelength spacing^1,2^. They present the most classic yet the most advanced form of coupled subwavelength resonators, and have extended the application range to the visible region^3^. Phase-gradient metasurfaces, which create arbitrary wavefronts using arrays of deep subwavelength antennas with unequal shapes, have given rise to a new frontier in metamaterials that had previously focused on three-dimensional bulk materials^4^. In these metasurfaces, the spacing between the antennas is smaller than the wavelength, but not too small to avoid strong near-field mutual coupling. Recently, through far-field mutual coupling, deep subwavelength resonators arranged with optimized wavelength-sized distances have demonstrated a coherent laser oscillation equivalent to a single, large, and high-power surface-emitting laser^5^.

Arrays of optical patch antennas^6^ have also been used for perfect absorbers^7^ and thermal emitters^8,9^. Optical patch antennas are subwavelength metal–insulator–metal (MIM) cavities that exhibit the half-wave resonance of the transverse-magnetic (TM) gap plasmon mode^10^. One significant potential benefit of an MIM cavity is that the upper and lower metal layers can also be used as electrodes. By sandwiching functional materials, strong interaction with densely confined electromagnetic fields could enable the realization of high-performance optoelectronic devices. To achieve this, individual patch antennas electrically isolated so far should be interconnected by conducting wires. Recently, patch antennas one-dimensionally (1D) linked by simple straight wires have remarkably enhanced the sensitivity of infrared detectors^11^, and even demonstrated gigahertz response at room temperature^12^. However, their polarization dependence distorted by the wiring revealed the lack of reasonable design principles for electrical connection of arrayed patch antennas, in contrast to the extensively investigated single dipole antennas^13,14^.

Herein, we propose a clear-cut architecture, synchronously wired antennas (SWAs), which are deep subwavelength arrayed resonators interconnected with geometrically engineered wires. We shed light on the optical functions of conducting wires between antennas. Metallic wires folded to provide optimum optical phase delay electrically connect (short-circuit) the patch antennas, without disrupting the optimized antenna resonance. In this Article, we not only clarify the physical foundations underlying the SWAs, but also present their application to mid-infrared photodetectors incorporating only a single quantum well (QW). Practical responsivity without polarization dependence at a predetermined wavelength in the fingerprint region, extending up to room temperature, is promising for spectroscopic applications^15–18^ and free-space data transfer^12^. However, applications are not limited to detectors; SWAs offer greater design freedom as a fundamental platform for optoelectronic metadevices^19–21^.

## Results

### Connecting antennas by conducting wires

We discuss optical patch antennas sandwiching GaAs/AlGaAs QW infrared photodetectors (QWIPs)^22,23^ with the sensitivity peak at λ = 6.7 μm between two Au layers. QWIPs absorb infrared light based on intersubband transitions (ISBT) and generate a photoconductive current by applying a bias voltage. The peak wavelength can be selected artificially by the QW design. However, QWIPs have two shortcomings. First, because of the ISBT selection rule, QWIPs can absorb only the vertical component of electric field relative to the QWs (*E*_*z*_)^24^. Thus, there is no sensitivity to normal incidence, and techniques such as oblique incidence and diffraction gratings are required. Another drawback is the low absorption of ISBT compared to the interband transition. Both of these problems can be overcome by incorporating QWIPs into optical patch antennas. Patch antennas rotate the horizontal electric field, *E*_*x*_, of normal incidence to a vertical field, *E*_*z*_, through magnetic coupling (*H*_*y*_). Furthermore, the magnitude of *E*_*z*_ is resonantly enhanced by the confinement of the field to a small volume. Both effects essentially magnify the sensitivity^11,12,25^.

First, based on a numerical model study, we observe how the properties of arrayed optical antennas are manipulated by wiring. The thickness of the QWIP (semiconductor) layer, its effective index, and the thicknesses of the upper and lower Au layers herein are *T* = 200 nm, 3.05 + 0.03i, 100 nm, and 200 nm, respectively. These correspond to the experimental parameters that will be presented later.

**Fig. 1:**
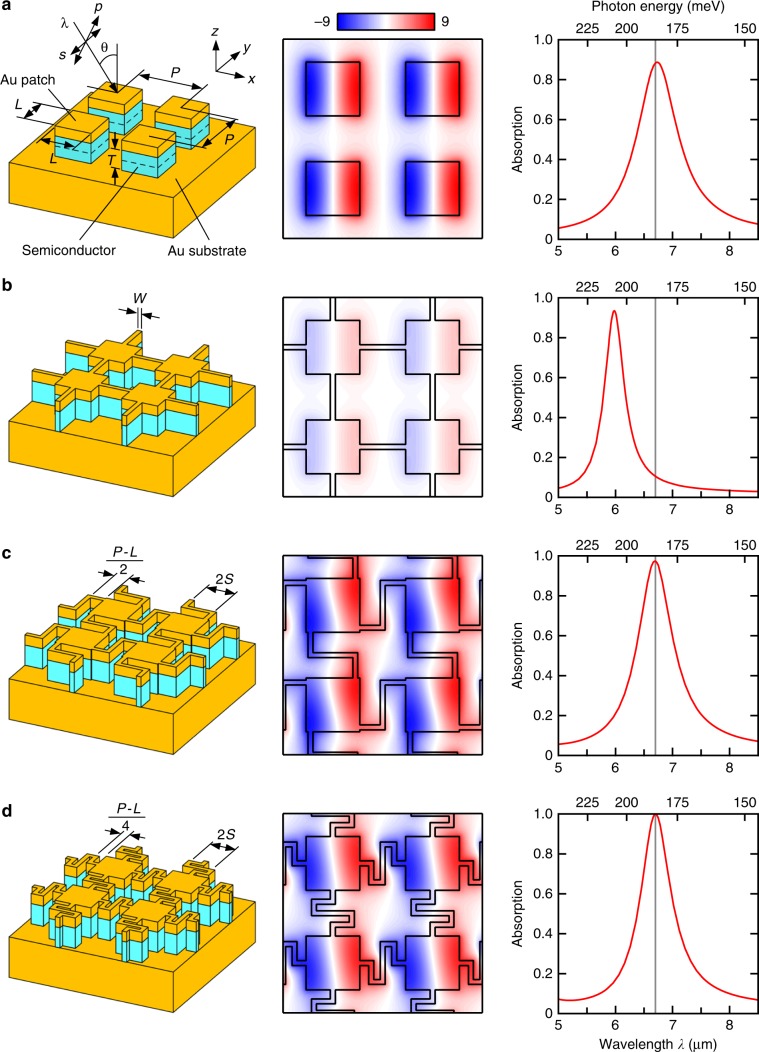
Structure (left), *E*_*z*_ distribution at λ = 6.7 μm (center) and absorption spectra (right) of the arrayed optical patch antennas. **a** Array of isolated antennas serving as the fundamental structure for this model study. **b**–**d** Antennas connected with **b** straight, **c** Z-shaped (*S* = 0.45 μm), and **d** S-shaped (*S* = 0.38 μm) wires. The schematics show the definitions of the coordinate system, incidence angle, polarization, and dimensions. *L* = 1.08 μm, *P* = 2.0 μm, *T* = 200 nm, and *W* = 100 nm. The vertical lines in the absorption spectra represent the sensitivity peak wavelength of QW (λ = 6.7 μm). The center column shows the snapshots of *E*_*z*_ at its maximum moment on the center *xy* plane of the semiconductor layer (broken lines in the schematic of **a**) for *x* polarization, θ = 0°; the value is normalized by the incident electric field.

An array of square antennas with a side length *L* = 1.08 μm (λ/6.2) and period *P* = 2.0 μm (λ/3.4) exhibits nearly perfect absorption at the QW sensitivity peak, irrespective of the polarization (Fig. 1a, right). Each antenna supports a half-wave resonance of *E*_*z*_ (Fig. 1a, center). *E*_*z*_ at both edges in the *x*-direction exhibits maximum amplitude but with the opposite polarity. The crucial requirements for a photodetector are maximizing the photocurrent (i.e., optical area) while minimizing the dark current (i.e., electrical area)^11,12,26^. The latter is particularly important for high temperatures, where the dark current dominates. Although the optical area can exceed the geometrical size of the antenna (up to ~*P*^2^)^27–29^, the electrical area is simply the geometrical size of the semiconductor layer. In the arrayed antennas discussed here (Fig. 1a, left), the semiconductor layer outside the Au patches is completely removed for minimizing the electrical area. Nevertheless, the resultant structure does not function as a detector, because there is no way for collecting the photocurrent. The most straightforward way to realize biasing and current extraction while minimizing the electrical area would be by connecting the patch antennas with thin wires (Fig. 1b, left). However, if linear wires with a width of *W* = 100 nm are added between the antennas, the electric field at the QW sensitivity peak reduces drastically (Fig. 1b, center). Because the resonator edges with opposite polarities are short-circuited by the conductive wires, the deterioration of resonance might seem to be natural. However, the resonance is not diminished, but is moved to another center frequency, even gaining a higher quality factor (Fig. 1b, right). This suggests that the wires are performing substantial functions as plasmonic waveguides. Here, we found that the original resonance can be restored by folding the wires to form a Z shape (crank shape) and adjusting their lengths, without enlarging the electrical area (Fig. 1c). Many similar solutions can be found; e.g., S-shaped wires but with different total lengths (Fig. 1d).

### Demonstration of antenna-enhanced infrared photodetectors

Optical patch antennas sandwiching QWIP layers, interconnected by folded wires, were fabricated and characterized. In addition to the SWAs, we incorporated two other important factors—a single QW and built-in ohmic contacts^30^.

The responsivity, *R*, of a QWIP is proportional to the absorption efficiency (internal quantum efficiency), η_abs_, and the photoconductive gain, *g*; *R* = *e*/(*h*ν) × η_abs_ × *g* = *e*/(*h*ν)  × η_conv_, where *e* is the electron charge, *h* is Planck’s constant, ν is the frequency, and η_conv_ *=* *g* η_abs_ is the conversion efficiency (external quantum efficiency). Here, the terminology of Schneider and Liu^23^ is followed. In a conventional QWIP, *R* is independent of the number of QWs, *N*_qw_, because η_abs_ ∝ *N*_qw_, but *g*  ∝ 1/*N*_qw_. However, the detectivity that defines the signal-to-noise ratio (SNR) is higher for larger *N*_qw_; thus, *N*_qw_ in the order of 10 has been preferred conventionally. Nevertheless, in a QWIP with vastly enhanced absorption by the cavities, *R* is inversely proportional to *N*_qw_ because η_abs_ no longer depends on *N*_qw_^11,12,23,29^. The dark-current-limited detectivity is also higher for smaller *N*_qw_. Therefore, we employed a single QW (*N*_qw_ = 1), which also leads to a higher field enhancement because of a thinner semiconductor layer.

Among the III–V semiconductor materials used for QWIPs, GaAs/AlGaAs is a well-established system. However, there is a drawback with GaAs regarding its electrical connection with a metal, i.e., alloying is necessary for a low-resistance ohmic contact. Alloyed interlayers are unacceptable for plasmonic devices that require smooth, abrupt, controlled metal–semiconductor interfaces. We realized the double-sided nonalloyed ohmic contacts to GaAs by optimizing the dopant density and growth temperature^30^. The ohmic contact to the originally buried surface particularly required the precise tuning of growth conditions. Now the low-resistance structure is epitaxially built-in; therefore, both the metal–semiconductor interfaces satisfy both electric and plasmonic requirements only by depositing metals.

**Fig. 2:**
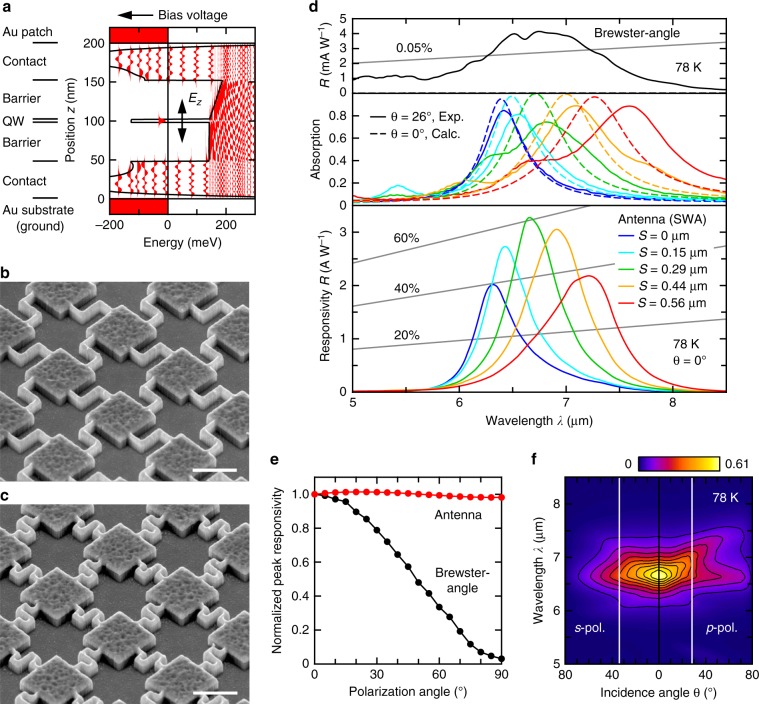
Fabrication and characterization of wired-antenna-enhanced QWIPs. **a** QW design, and numerical conduction band profile and wave functions based on experimental dopant density distribution. **b** Scanning electron micrograph of SWAs with Z-shaped (*S* = 0.29 μm) and **c** S-shaped (*S* = 0.22 μm) wires. Bar: 1 μm. **d** Responsivity spectrum of the reference Brewster-angle detector (top), absorption spectra for Z-shaped SWAs with various *S* values measured for θ = 26°, *s*-polarized incidence (solid lines, middle) and calculated for θ = 0° (broken lines, middle), and the corresponding responsivity spectra for θ = 0° (bottom). Equiefficiency (η_conv_) lines are also plotted. **e**, **f** Other properties for the detector with the maximum responsivity (Z-shaped, *S* = 0.29 μm). **e** Polarization angle dependence of *R*_p_. Results for the Brewster-angle detector are also shown for reference. **f** Incidence-angle dependence of η_conv_ for *p*- (right) and *s*-polarization (left). Vertical lines denote the FOV/2 at half height.

The QW was designed based on the standard bound-to-continuum structure^22^. The details of design, fabrication, and characterization of the specimens are given in Methods. The temperature of the detector is 78 K, unless specified otherwise. The QWIP layer (*T* = 200 nm) was made of a 4-nm-thick GaAs QW sandwiched by 50-nm Al_0.3_Ga_0.7_As barrier layers and 48-nm GaAs built-in ohmic contact layers (Fig. 2a). The quantum structures were fabricated by molecular beam epitaxy on n-GaAs substrates. Before incorporating into SWA structures, we fabricated a Brewster-angle incidence detector as a reference detector, to confirm the intrinsic properties of the QWIP layer. A peak unpolarized responsivity of *R*_p_ = 4.1 mA W^−1^ at 6.73 μm (Fig. 2d, top), consistent with the design, was obtained.

The wafer transfer technique is indispensable to the incorporation of epitaxial semiconductor layer into the optical patch antennas^31–33^. The QWIP layer was transferred to another GaAs substrate through Au–Au diffusion bonding, and was sandwiched between Au layers by patterning Au/Ti SWA structures on the top using electron-beam drawing. Finally, the GaAs/AlGaAs layers other than the antennas and wires were removed by vertical dry etching, to minimize the electrical area. To investigate the effects of wire geometries on the photodetector performances systematically, SWAs with various wire folding shapes and lengths, *S*, were fabricated. Representative wire geometries are shown in Fig. 2b, c, and Supplementary Fig. 1b (see also Supplementary Note 1).

The absorption and normal-incidence responsivity spectra of Z-shaped SWA detectors with several different *S* values are shown in Fig. 2d (middle and bottom; note that *S* = 0 corresponds to straight wires). The results for *L* = 1.19 μm rather than 1.08 μm discussed in Fig. 1 are shown because higher responsivities and detectivities were obtained despite the increased dark currents; suggesting a fair balance of the optical and electrical areas. However, the systematic dependence on *S* values is identical irrespective of *L* values.

Because the experimental absorption spectra (solid lines) could only be obtained for oblique incidence by reflection measurement, the numerical absorption spectra for θ = 0° are presented for reference (broken lines). In this calculation, the semiconductor layer was exactly treated as a multilayer made of a QW, two barrier, and two contact layers, considering the free-carrier absorption and uniaxial ISBT absorption. In both absorption spectra, the peak systematically moves to the longer side for a larger *S*. The responsivity peak wavelengths elongate simultaneously within the sensitivity range of the Brewster-angle detector (3–8 μm). When the absorption peak of the SWAs for θ = 0° (Fig. 2d, middle, broken lines) and the responsivity peaks of the QWIP layer (Fig. 2d, top) coincide (*S* = 0.29 μm), the maximum responsivity *R*_p_ = 3.3 A W^−1^ (η_conv_ = 0.61) was recorded at 6.67 μm (Fig. 2d, bottom). The responsivity was enhanced by a factor of 803 compared with that of the Brewster-angle reference detector. To estimate the theoretical responsivity from the numerical absorption, the value of *g* is necessary. The spectral density of the dark-current noise^34^ gave *g* = 2.3 (Supplementary Fig. 2a), which yields good agreement between the theoretical and experimental responsivities (Supplementary Fig. 1e). The detectivities determined from dark-current and background (298 K) noises are 5.2 × 10^10^ and 3.9 × 10^10^ cm Hz^1/2^ W^−1^, respectively. Thus, our detector is in a background-limited regime at 78 K. The noise-equivalent power (background incidence) is 0.26 pW Hz^−1/2^.

There is no polarization dependence (Fig. 2e) due to the symmetry of SWAs. The angular dependence of η_conv_ reveals that the field of view (FOV) of the detector is relatively narrow (57° and 68° for *p* and *s* polarizations, respectively; Fig. 2f and Supplementary Fig. 1g) compared with that of the conventional unstructured detectors (120° in principle). This is because the dispersive nature of the wiring (discussed later) limits the overlapping of sensitivity range of the QW and the antenna resonance. An intrinsically small FOV is advantageous for an infrared detector in that it suppresses the photocurrent due to background radiation from unwanted directions.

**Fig. 3:**
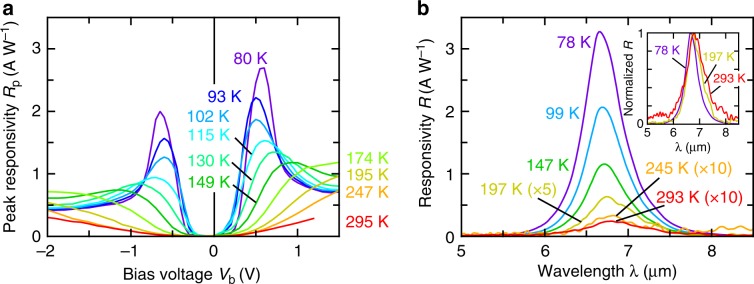
Temperature dependence of the responsivity of wired-antenna-enhanced QWIPs. Z-shaped, *S* = 0.29 μm. **a** Bias dependence of *R*_p_ at various detector temperatures, measured with a blackbody at 500 °C. **b** Temperature dependence of spectroscopic responsivity at their respective peak bias voltages. For 197 K, 245 K, and 293 K, the spectra were obtained at *V*_b_ = +0.5 V, as no peak could be specified. The inset shows the normalized spectra at representative temperatures including the one at room temperature (293 K).

The bias voltage, *V*_b_, dependence of *R*_p_ measured using a 500 °C blackbody^22^ at various detector temperatures is shown in Fig. 3a. The *R*_p_ is not linear with *V*_b_, but quickly increases after an offset and exhibits a peak at a certain value, which is consistent with previous reports for *N*_qw_ = 1^35,36^. The responsivity was observed up to room temperature (*R*_p_ = 24 mA W^−1^ at *V*_b_ = + 0.5 V, 293 K), and the peak wavelength exhibited a small red shift for a higher temperature (Fig. 3b). The background-limited performance (BLIP) temperature, specifying the operation range of a photon detector uninfluenced by the dark current, was 87 K (Supplementary Fig. 2c).

Previously, room-temperature sensitivity of photoconductive QWIPs had been observed only for intense laser sources^37^. Recent antenna-enhanced QWIPs^12^ with *N*_qw_ = 5 demonstrated the first room-temperature response for a blackbody emitter at 1000 °C, in addition to η_conv_ = 12.4% (*R*_p_ = 0.9 A W^−1^ at λ = 9 μm) and background-limited detectivity of 1.5 × 10^10^ cm Hz^1/2^ W^−1^ at 78 K. In the present work, using polarization-independent SWAs and *N*_qw_ = 1 realized higher performances (61% and 3.9 × 10^10^ cm Hz^1/2^ W^−1^ at 78 K), and room-temperature sensitivity for a blackbody at a lower temperature (500 °C). Although a straightforward comparison is difficult because of the difference in the peak wavelengths and FOVs for background measurement, our SWA-enhanced QWIP is one of the best GaAs-based mid-infrared photodetectors. This was achieved by enhancing the weak response of a single QW with SWAs (see Supplementary Fig. 3 and Supplementary Note 2 for detailed discussion).

### Resonance engineering based on wire geometry

As has been shown, numerical simulation offers accurate optical properties of SWAs with arbitrary wire geometry, but does not explain why the folding shape and lengths of wires control the resonance of SWAs. Through simple models, here we clarify the optical functions of the wires. Only the key findings are presented here, while the detailed calculation method and discussions are presented in Methods and Supplementary Notes 3 and 4.

Resonance is excited by the coupling of incident light with the in-plane propagation modes. For simplicity, the discussion is restricted to propagation in the *x*-direction by the *p*-polarized incidence in the *xz* plane, which excites the TM modes. In-plane wave vector in SWAs, *k*_*x*_, is discussed.

**Fig. 4:**
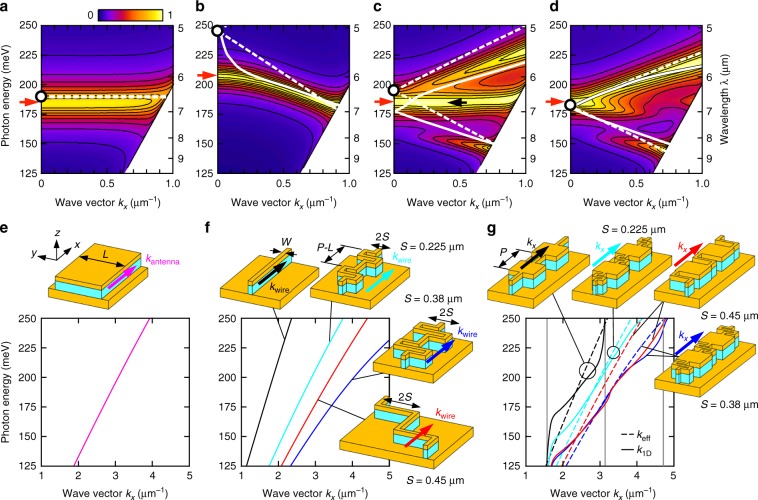
Resonance engineering of arrayed antennas based on wire geometry. **a**–**d** Wave-vector dependence of absorption spectra for *p*-polarized incidence. **a** Array of isolated antennas. **b** Antennas connected with straight, **c** Z-shaped (*S* = 0.45 μm), and **d** S-shaped (*S* = 0.38 μm) wires. **e**–**g** Dispersion relation (real part) of TM-like propagation mode in elementary structures illustrated by the schematics. **e** MIM waveguide with width *L*. This dispersion gives the resonance of arrayed isolated antennas represented in **a** by a broken line. **f** Infinitely repeated folded plasmonic wires. **g** 1D periodic system made of antennas and wires, *k*_1D_ (solid curves). The broken lines are *k*_eff_ based on Eq. (2). The dispersions of wired antennas represented by solid and broken lines in **g** are also displayed by solid and broken lines in **b**–**d**. The vertical gray lines denote the BZ edges where bandgaps can appear.

The numerically obtained *k*_*x*_ dependence of the absorption spectra of SWAs corresponding to Fig. 1a–d is shown in Fig. 4a–d as color maps, respectively. The resonance wavelengths for normal incidence (θ = 0°) presented in Fig. 1 appear as peaks at *k*_*x*_ = 0 (red arrows). The array of isolated antennas exhibits a flat mode (Fig. 4a); however, it is fundamentally modified when wired. Marked angular dispersion arises and the resonance wavelength changes, both of which greatly depend on the folding shape and length of wires (Fig. 4b–d). Our aim here is to reproduce these angular dispersions by a simple, insightful model.

Most features can be explained by the propagation properties of individual building blocks—antennas and wires. Numerically obtained wave vectors, *k*_antenna_ and *k*_wire_, of the TM-like plasmon mode of an infinitely long MIM waveguide of width *L* and infinitely repeated units of folded wires with a length, *P*−*L*, are shown in Fig. 4e, f, respectively, in an extended zone scheme.

An MIM waveguide of width *L* (Fig. 4e) supports a propagating mode close to the infinitely wide waveguide^38,39^. A square patch antenna of side length *L* is an MIM waveguide of width *L* terminated at a length *L*. Its resonance wavelength is given by

1 $${\mathrm{{Re}}}\left({k_{{\mathrm{{antenna}}}}} \right) \times L = \pi ,$$

where Re denotes the real part. Because the resonance is localized at each antenna, the same condition holds true for the resonance of arrayed isolated antennas. The dispersion deduced from Eq. (1) is superimposed with a broken line in Fig. 4a. Both the resonance wavelength (circle) and wave-vector dependence are well described herein.

The dispersion of wires (Fig. 4f, see definition in “Methods” section) varies in a wide range depending on the geometry; this variety gives the foundation of the high degree of design freedom of SWAs. The value of *k*_wire_ at a fixed photon energy (wavelength) depends both on the physical length of the wire and the folding shape. The former is natural because the phase rotates more while traveling unit length *P*−*L* in the *x* direction. Besides, the speed of the propagating plasmon wave varies depending on its location along the wires, particularly around the winding parts in the S-shaped wires (Supplementary Figs. 4 and 5). As a consequence, the accumulated phase rotation spanning unit length *P*−*L* in the *x*-direction depends on the folding shape. As expected, intermediate-shaped wires yield intermediate properties (Supplementary Note 5 and Supplementary Fig. 6).

The effective wave vector of a combined system of wires and antennas can be given by

2 $$k_{{\mathrm{{eff}}}} = k_{{\mathrm{{antenna}}}} \times L/P + k_{{\mathrm{{wire}}}} \times \left({P - L} \right)/P,$$

where the phase jump at the wire–antenna interface was ignored. Because *k*_wire_ is included here, the wired antennas exhibit a resonance wavelength essentially different from that of the arrayed isolated antennas. In Fig. 4g, *k*_eff_ is represented by broken lines. In Fig. 4b−d, the same results are shown by white broken lines in a reduced zone scheme. The resonance wavelengths for θ = 0° are given by Re(*k*_eff_) × *P* = 2π and denoted by circles. This simple model gives a sound description of the rigorous results in Fig. 4c, d.

However, the result for straight wires in Fig. 4b needs a more accurate model. We discuss a 1D array of wires and antennas, where the mismatch at the wire–antenna interface is considered. The obtained wave vectors, *k*_1D_ (see definition in “Methods” section) are represented in Fig. 4g by solid lines. *k*_1D_ deviates from *k*_eff_ near the Brillouin zone (BZ) edges (vertical lines). This implies the formation of bandgaps by reflection, i.e., impedance mismatch, at the wire–antenna boundaries (the bandgap is blurred by absorption. See Supplementary Note 4 for remarks). The white solid lines in Fig. 4b−d represent *k*_1D_ in a reduced zone scheme. This time, the model describes the rigorous result for the straight wire (Fig. 4b) for the most part (except for the vertical tail due to the bandgap). The discussion thus far would be sufficient to understand that the plasmonic propagation through the wires determines the resonance of SWAs.

In summary, (i) the conducting wires provide optical phase delay between the antennas depending on the folding shapes and dimensions, and (ii) the reflection at the wire–antenna interface also influences the overall properties of SWAs.

The residual discrepancies, e.g., the horizontal mode denoted by a black arrow in Fig. 4c, originate from the coupling in the *y*-direction (see Supplementary Movie 1 and Supplementary Note 4).

## Discussion

In summary, we demonstrated that an overlooked factor—the geometry of wires—functions as a new design freedom for tuning the resonance wavelength of arrayed antennas while minimizing their electrical areas, and enhances the infrared responsivity of QWIPs. Optical patch antennas sandwiching a QWIP interconnected with folded wires achieved a polarization-independent conversion efficiency exceeding 60% and room-temperature sensitivity for a 500 °C blackbody, when the absorption wavelength of the QW and the resonance wavelength of the wired antennas agree with each other.

Despite these excellent properties, the detectors have not been optimized fully yet. Antenna designs balancing the optical and electrical areas should be thoroughly investigated. The layer configuration of the QWIPs also needs improvement to maximize the ISBT absorption. QWIPs made of other materials (e.g., InGaAs/InP)^40^ have shown similar or higher responsivity and detectivity; SWAs can be combined with these materials as well. Through these optimizations, less toxic SWA-enhanced QWIPs would replace the widely used but toxic HgCdTe detectors^26^ in some spectroscopic applications^15–18^. As Palaferri et al. demonstrated^12^, gigahertz-frequency detection would be another promising application that only antenna-enhanced QWIPs can realize.

We stress here that all the structures, both the quantum nanostructures manipulating the electron wave functions and the antennas controlling the electromagnetic waves, were artificially created and yielded quantitatively reasonable properties (see Supplementary Notes 6 and 7). Such highly engineered structures suggest a novel and important frontier in optoelectronics. SWAs can also be applied to various semiconductor structures other than QWIPs to realize thin photodetectors with high absorption ideal for reducing dark currents^26,41^. Moreover, SWAs are expected to serve as a fundamental architecture of arrayed subwavelength resonators for optoelectronic metadevices such as solar cells, emitters, and modulators^19–21^.

SWA and three other types of arrayed subwavelength resonator introduced at the beginning are compared in Supplementary Note 8 and Supplementary Fig. 7. In a conventional array, the resonators are mutually coupled through propagation in free space, making the coupling straightforwardly dependent on the relative positions of the resonators. By contrast, in SWAs, the phase relationships can be varied widely by controlling the wire folding geometry independent of the relative antenna positions. This gives us greater freedom when designing metasurface optoelectronic devices.

Before finishing, we remark that each antenna or wire in SWAs does not have to be identical. By changing the wires and/or antennas gradually, the wavefront engineering that has been achieved using phase-gradient metasurfaces could be expanded further with wider tunability. Supplementary Note 9 and Supplementary Fig. 8 exemplifies a design of an SWA-enhanced QWIP with a high responsivity to a specific wavefront by incorporating gradually changing wires.

## Methods

### Design, fabrication, and evaluation of quantum wells

The QWIP structure was grown on n-GaAs (100) substrates (Si density: 1 × 10^18^ cm^−3^) using a solid-source molecular beam epitaxy system. After the growth of a GaAs buffer layer, sacrificial (etch stop) layers of Al_0.9_Ga_0.1_As (thickness: 900 nm, Si density: 5 × 10^17^ cm^−3^) and Al_0.55_Ga_0.45_As (100 nm, 5 × 10^17^ cm^−3^) were grown. Then, an initial-side contact layer with a total thickness of 48 nm^30^, consisting of a highly Si-doped GaAs layer (28 nm, 1.25 × 10^19^ cm^−3^), an n-GaAs (15 nm, 3 × 10^18^ cm^−3^), and a nondoped GaAs layer (5 nm), was grown. The main part of the QWIP is made of an Al_0.3_Ga_0.7_As barrier (50 nm), an n-GaAs QW (4 nm, 3 × 10^18^ cm^−3^, except for the final 0.85 nm), and an Al_0.3_Ga_0.7_As barrier (50 nm). Finally, a 48-nm-thick end-side contact layer comprising an n-GaAs (20 nm, 3 × 10^18^ cm^−3^) and a highly Si-doped GaAs layer (28 nm, 1.25 × 10^19^ cm^−3^) was grown. To form the highly Si-doped GaAs layers, the growth of n-GaAs (28 nm, 5 × 10^18^ cm^−3^) was interrupted and seven δ-doping layers of Si (3 × 10^12^ cm^−2^) with a period of 4 nm were inserted. The layers from the initial-side contact to the end-side contact conform the QWIP layer with a total thickness of *T* = 200 nm. For this QWIP layer, the growth temperature was kept at relatively low 530 °C, which is important for realizing nonalloyed ohmic contact to the initial side.

The designs of the QW and the barriers are based on the standard bound-to-continuum structure^22^. In the etch-stop layer, there are two layers in the Al composition. The first Al_0.9_Ga_0.1_As layer is for high etching selectivity against the GaAs substrate and the next Al_0.55_Ga_0.45_As finishing layer is for realizing a smooth, low-loss surface^42^. For responsivity evaluation by the Brewster-angle detector before the wafer transfer, the etch-stop layers are also doped.

The depth profile of the doped Si concentration was measured with secondary-ion mass spectrometry. The total active carrier sheet density was confirmed with the van der Pauw method for specimens made of only the QWs or contact layers^30^.

The conduction band profiles, wave functions, and absorption spectra were numerically obtained by solving the Schrödinger and Poisson equations^43^ and using Fermi’s golden rule^24^. Conduction band offset was based on ref. ^44^. However, a small correction was applied on the basis of systematic experiments; Δ*E*_c_ = 0.64 Δ*E*_g_ was adopted at 78 K, where Δ*E*_c_ and Δ*E*_g_ are conduction band offset and bandgap difference, respectively.

The asymmetrical doping profile including a 5-nm-thick nondoped layer in the initial-side contact layer is employed, aiming at obtaining a symmetrical band profile after the segregation of the Si dopant. However, this attempt was not successful. Hence, the profile obtained was asymmetric, as shown in Fig. 2a. The asymmetry also appeared in the current–voltage properties in Supplementary Fig. 2.

Brewster-angle detectors were fabricated from a small piece of wafer. After evaluating the intrinsic responsivity of the QWIP layer, the wafer transfer was performed and the antenna-enhanced detectors were fabricated.

Because the QW was fabricated on a highly doped substrate, the measurement of the absorption spectrum by transmission was difficult. Therefore, the absorption spectrum was obtained from the responsivity spectrum of the Brewster-angle detector and the value of *g* (= 3.0, explained in Supplementary Note 1) obtained from the noise measurement. The absorption spectrum yielded an oscillator strength of 0.74 and a dipole matrix element of 1.5 nm.

### Electromagnetic simulation and design of antennas

For the numerical simulation based on Maxwell’s equations, several different methods were used. The numerical results shown in this paper were obtained by finite difference methods (COMSOL Multiphysics). In the early stage of this study, rigorous coupled-wave analysis (RSOFT, DiffractMOD) and finite-difference time-domain method (RSOFT, FullWAVE) were also used.

The dielectric function of Au was based on ref. ^45^. For Figs. 1 and 4 and Supplementary Figs. 4–6, and 8, where reflection and propagation of the overall structures are important rather than the accuracy of the electric field at the QW layer, the semiconductor QWIP layer was regarded as an isotropic wavelength-independent medium with a refractive index of 3.05 + 0.03i, for simplicity. This value gives practically consistent results with the rigorous five-layer model.

For the rigorous analysis shown in Fig. 2 and Supplementary Figs. 1 and 3, and the Fresnel equations for the Brewster-angle detector, where an accurate vertical electric field at the QW layer is necessary for obtaining responsivity, the QWIP layer was treated as a wavelength-dependent five-layer multilayer. The QW was assumed as a uniaxial material with ISBT absorption in the vertical direction and free-carrier absorption in the lateral directions.

The wavelength-dependent refractive indices of the nondoped GaAs and Al_0.3_Ga_0.7_As were based on ref. ^46^. To express the free-carrier absorption, a Drude term −ω_p_^2^/(ω^2^ + iγω) was added, where ω_p_ and 1/γ are the plasma frequency and relaxation time, respectively^47^, both of which are specified by the carrier density, *N*, and mobility, μ, respectively^24^. The contact layer was regarded as an isotropic Drude material corresponding to experimentally obtained *N* = 4.7 × 10^18^ cm^−3^ and μ = 1500 cm^2^ V^−1^ s^−1^. For the lateral directions of the QW layer, a Drude term corresponding to the experimental *N* = 2.4 × 10^18^ cm^−3^ and μ = 2000 cm^2^ V^−1^ s^−1^ was considered. In the vertical direction of the QW layer, the ISBT absorption was described with a three-term Lorentzian model^27^, considering the term −Ω_pj_^2^/(ω^2^ − Ω_j_^2^ + iΓ_j_ω) (*j* = 1, 2, and 3). We set (Ω_1_, Γ_1_, Ω_p1_) = (168, 30, 256), (Ω_2_, Γ_2_, Ω_p2_) = (236, 39, 114), and (Ω_3_, Γ_3_, Ω_p3_) = (279, 73, 124) in meV. These values were obtained initially by Fermi’s golden rule^24^ and then by making a small correction to *j* = 1, so that the values yielded consistent results with the experimental responsivity of the Brewster-angle detector.

The definitions of the geometry of SWAs are given in Fig. 1 and Supplementary Fig. 6. In the polarization dependence in Fig. 2e, the angle 0° corresponds to the *p*-polarization (*x* polarization for θ = 0°). Exactly, the Z-shaped wires with *S* = 0.56 μm shown in Fig. 2d and Supplementary Fig. 1e have a slightly different definition, because the *S* value is so large that the wires do not fall within the side length *L*. In such situations, the geometry in Supplementary Fig. 1c with *F* = 0.05 μm was used.

In Fig. 4 and Supplementary Figs. 4–6, to determine the in-plane propagation of the wires, *k*_wire_, and wire/antenna combinations, *k*_1D_, five period structures were considered. An oscillating *E*_*z*_ was placed at the wire edge and excited TM-polarized in-plane propagating modes. An entrance straight wire with *W* = 0.1 μm and a proper length was added at specific instances to stabilize the incident electromagnetic fields. The outer area of this model was terminated by perfect matching layers, so the model was equivalent to an infinitely repeated folded wire.

The definition of *k*_wire_ determined by numerical simulation needs a special remark. Here we are not discussing a plane wave propagating in a free space. Instead, we are considering a guided wave propagating through a deep subwavelength structure with a width *W* = 0.1 μm (< λ/60) folded at a right angle several times over within a length of *P*−*L* = 0.92 μm (< λ/7). The focus here is on how far the phase evolves before the guided wave arrives at the next square cavity placed at a distance of *P*−*L* in the *x*-direction. To determine this, the same wire units with a length of *P*−*L* in the *x*-direction were connected (see the schematics in Fig. 4f and Supplementary Fig. 6c), and the average phase rotation was numerically obtained with respect to the distance in the *x* direction. This is called *k*_wire_.

The definition of *k*_1D_ is similar to *k*_wire_. In the case of *k*_1D_, the unit structure is made of one unit of wire with a length of *P*−*L* in the *x-*direction and an MIM waveguide with width *L* and length *L* (see the schematics in Fig. 4g and Supplementary Fig. 6d). A TM-polarized wave is excited at the edge of the first wire and the average phase rotation with respect to the distance in the *x*-direction was numerically obtained.

### Fabrication of Brewster-angle detectors

The intrinsic properties of the QWIP layer were evaluated by a Brewster-angle incidence detector. The Brewster-angle configuration exhibits a lower responsivity than the widely used back-illumination through a 45° facet, but its fabrication is easier. A Ti (bottom)/Au (top) electrode with a 100 μm × 400 μm aperture was patterned on top of a mesa defined by a H_2_SO_4_;H_2_O_2_:H_2_O solution (1:8:1,000). The long rectangular shape is because the light is incident from a very shallow angle. Due to the built-in ohmic layer, this electrode exhibits a low resistance only by depositing the metals. However, the substrate side needs alloying for the ohmic contact. An electrode made of Ni, AuGe, Ni, and Au was patterned on a wet-etched substrate surface and annealed at 420 °C for 90 s. Although the exact Brewster angle is 73°, the detector was set at θ = 65° in the actual experiment due to the restriction of the optical setup. This actual value was used in the calculation. The setup for the evaluation of the Brewster-angle detector is shown in Supplementary Fig. 1d. This detector has a sensitivity only for *p*-polarized incidence. In the polarization dependence in Fig. 2e, the angle 0° corresponds to the *p*-polarization. The definition of the sign of bias voltage is opposite to the antenna-enhanced detector, because the QWIP layer is upside down.

### Wafer transfer

The QWIP layer was coated with 3-nm-thick Ti and 150-nm-thick Au layers and bonded with another n-GaAs substrate coated with 10-nm-thick Ti and 500-nm-thick Au layers at a pressure of 5 MPa and a temperature of 250 °C for 60 min in N_2_ atmosphere^48^. After mechanical polishing, the original GaAs substrate was chemically etched by a citric acid (1 g ml^−1^):H_2_O_2_ solution (10:1) at 38 °C^49^. By etching the etch-stop AlGaAs layers using HF, the 200-nm-thick epitaxial QWIP layer was transferred onto an Au substrate.

### Fabrication of antenna-enhanced detectors

Wired antennas were fabricated on the transferred QWIP layer by electron-beam drawing and lift-off of 3-nm-thick Ti and 150-nm-thick Au top layers. Most of the QWIP layer was removed using a H_2_SO_4_;H_2_O_2_:H_2_O solution (1:8:1000), leaving only the minimum necessary areas around the antennas. Then, the specimen was coated with a 100-nm-thick SiO_2_ film for electrical isolation. Windows of the SiO_2_ layer were opened by dry etching using SF_6_ around the detector areas. SF_6_ was chosen because of its good selectivity against GaAs and excellent removal of SiO_2_ on the vertical side wall of the upper Au layer. After patterning the electrode pads made of 3-nm-thick Ti and 150-nm-thick Au layers, so that one side can overlap with the edges of the wired-antenna areas, the QWIP layers not covered by the upper Au layers were vertically etched by inductively coupled plasma etching using Cl_2_ and N_2_. The resultant Au layer after the dry etching was a ~100 nm in thickness. For the patterning of the structures, except for the first antennas, the laser-beam drawing method was used. Each detector is a 100 μm square and contains 2500 patch antennas. The global image of a detector is exemplified in Supplementary Fig. 1a.

For efficient systematic evaluation, seven such detectors with different parameters were integrated on a chip and assembled on an 8-pin ceramic package. Another pin is for the common ground. The signals were extracted by bonding an Au wire at the root of each Au electrode.

### Measurement of optical properties

The detector was installed in a liquid-N_2_ cryostat with ZnSe windows, and the responsivity spectra were measured with a Fourier transform infrared (FTIR) spectrometer (JASCO, FT/IR-6200). The current from the detector was amplified (NF, CA5350 and Keithley, 428, depending on the experiment) and fed into an external port of the FTIR. The spectral responsivity was quantified based on a calibrated HgCdTe detector. No corrections other than the detector areas were applied to the responsivity values. The incident light from the FTIR was unpolarized. When necessary, a wire-grid polarizer was placed in the light path. The ceramic package mounting the detector chip was in tight contact with a Cu block. The specimen in the cryostat was made to rotate around its vertical axis so that the incidence-angle dependence could be evaluated.

In addition to the spectroscopic measurement, the measurement of *R*_p_ based on the method described in ref. ^22^, by the use of a blackbody light source at 500 °C, was conducted to observe the overall dependence on *V*_b_ and detector temperature. The radiation was incident on the detector through a chopper, and the photoconductive current was measured with a lock-in amplifier (SRS, SR830).

The reflection spectra were measured with an infrared microscope equipped with a Cassegrain objective lens connected to the FTIR. A relatively small incidence angle distribution centered at 26° was realized with an aperture.

### Measurement of electrical properties

To measure the dark current, the detector was covered by a cold shield with a blackbody coating cooled at the same temperature as the detector (e.g., 78 K). When the cold shield is removed, a background light at 298 K is incident on the detector from an area with an effective FOV of 102°. The current–voltage relationship was measured with a source meter (Keithley, 2635B).

To determine the values of dark-current-limited detectivity and *g*, the power spectral density of the amplified current was measured with a fast Fourier transform analyzer (Ono Sokki, CF-4700). The value of *g* (Supplementary Fig. 2a) was determined from the dark current, *I*_dark_, and noise spectral density *i*_n_/Δ*f*^1/2^ (Δ*f*: bandwidth) by the relationship *i*_n_/Δ*f*^1/2^ = (4*egI*_dark_)^1/2^. The noise spectra exhibited a plateau-like region in the range of 0.5–3 kHz. In this study, the value at 1 kHz was used^34^.

## Supplementary information

Supplementary Movie 1

Supplementary Information

Description of Additional Supplementary Files

## Data Availability

The authors declare that the data supporting the findings of this study are available within the paper and its Supplementary information files.
