# Supplementary material for: Synchronously wired infrared antennas for resonant single-quantum-well photodetection up to room temperature
Source: Nat Commun. 2020 Jan 28;11:565. doi: 10.1038/s41467-020-14426-6 (PMC6987185; doi:10.1038/s41467-020-14426-6)
Supplement: Supplementary file 2 — Supplementary Information [file 41467_2020_14426_MOESM2_ESM.pdf]

Synchronously wired infrared antennas for resonant single-quantum-well photodetection up to room temperature

Miyazaki et al.

Supplementary Note 1. Detailed discussion on detector performances

In the global view of a detector shown in Supplementary Fig. 1a, the area colored light blue is covered by SiO_2. Au patches are connected to the upper Au electrode, but isolated from the Au substrate (yellow) by the QWIP layer.

Because only the results for the Z-shaped SWAs with L = 1.19 μm could be presented in the main text, the responsivity spectra measured for other representative detectors are shown in Supplementary Fig. 1f, along with the corresponding calculation results (broken lines). The agreement between the experiment and calculation is fairly good. The Z-shaped SWAs with L = 1.08 μm discussed in Figs. 1 and 4 were actually fabricated, but their responsivity and detectivity were inferior to those of Z-shaped SWAs with L = 1.19 μm. The properties of the detectors with L = 1.08 μm (S = 0.46 μm) at 78 K were as follows; responsivity: 2.5 A W^{-1}, dark-current- and background-limited detectivities: 4.7×10^{10} and 3.1×10^{10} cm $Hz^{1/2}$ W^{-1}, respectively. However, the detector with L = 1.08 μm exhibited a BLIP temperature, T_{BLIP}, of 90 K, which is higher than that for L = 1.19 μm. This suggests that T_{BLIP} is straightforwardly determined by the electrical area as discussed in Refs. 1 and 2.

The incidence-angle dependence of η_{conv} obtained by numerical simulation of Z-shaped SWAs with S = 0.29 μm corresponding to Fig. 2f is shown in Supplementary Fig. 1g. There is an excellent agreement between the experimental measurements and the calculation, including

small features. Supplementary Fig. 1h illustrates the experimental incidence-angle dependence for S-shaped SWAs with L = 1.19 μm and S = 0.22 μm. The experimental FOVs for the p and s polarizations are 60° and 97°, respectively. Supplementary Fig. 1i is that of the intermediate-shaped SWAs with L = 1.19 μm and S = 0.29 μm (Supplementary Note 5), and the FOVs for the corresponding p and s polarizations are 45° and 77°, respectively. Only for the angle dependence does the intermediate-shaped structure exhibit the sharpest result, which suggests the highest insensitivity to the background photon noise.

The R_p in Fig. 3a measured using a blackbody emitter and spectroscopically measured with an FTIR in Fig. 3b exhibited fair agreement despite their very different methods. However, in the temperature range higher than 250 K, the spectroscopic R_p (Fig. 3b, by FTIR) exhibited lower values by a factor of two. In this high temperature range, a condensing optics was added in front of the detector to enhance the photocurrent. It is assumed that the R_p was underestimated due to the different incidence-angle dependence of the reference HgCdTe detector and that of our detector (wide and narrow, respectively). However, we can conclude that the room-temperature responsivity of our detector is at least 24 mA W^{-1}, which was evaluated with the FTIR.

Supplementary Fig. 2a shows g determined from the dark-current noise for the Brewster-angle detector. The antenna-enhanced detector showed a very similar result. Thus, g is unique

to the QWIP layer. The V_b dependence of R_p in Fig. 3a is very similar to that of g, which is natural because $R_p \propto g$. The peak V_b value for R_p of the Brewster-angle detector and that for the peak in g were consistent; g = 3.0 was recorded at V_b = +0.45 V for the Brewster-angle detector. However, the V_b values showing the highest responsivity of antenna-enhanced detectors were always higher than those for the Brewster-angle detectors by 0.1 V. The reason for this is not yet clear. For the Z-shaped SWAs with L = 1.19 μm and S = 0.45 μm that gave the maximum responsivity, g was 2.3 at V_b = +0.55 V.

The photoconductive gain can be expressed as $g = \tau_c v/(N_{qw}L_p)$, where τ_c and v are capture time and drift velocity, respectively. Since we have only a single period, L_p is taken as the total length of the barriers and the well[3]. From similar structures, τ_c = 5 ps[3] and $v = 5\times10^6$ cm s^{-1} (Ref. 2) at 78 K can be estimated, and by using N_{qw} = 1 and L_p = 104 nm, g = 2.5 is obtained, which is consistent with the experimental value (2.3–3.0).

Supplementary Fig. 2b shows the V_b dependence of the dark-current density, J_{dark}, at various temperatures. Supplementary Fig. 2c displays the ratio of the background-incident current, J_{298K}, with respect to the dark current. T_{BLIP} is defined as the temperature when this ratio exceeds two (SNR exceeds one).

In this study, many properties have exhibited asymmetric V_b dependence, which is natural due to the asymmetric dopant distribution. Because the positive V_b region yielded

higher responsivity and detectivity, the properties in the positive region were discussed mainly; e.g., T_{BLIP} (87 K). The high g at $V_b > 0$ suggests a higher τ_c for this direction. On the other hand, the negative V_b region yields a higher J_{298K}/J_{dark}, suggesting T_{BLIP} = 90 K. This happens because the J_{dark} at $V_b < 0$ is one order of magnitude smaller than that at $V_b > 0$.

Supplementary Note 2. How does a single thin QW harvest the incoming light efficiently?

Based on the numerical simulation, we discuss how a 4-nm-thick single QW absorbs the incident light in the maximum-sensitivity structure (Z-shaped, S = 0.29 μm). Here, the semiconductor is treated strictly as a five-layer multilayer. Supplementary Fig. 3a confirms that the horizontal electric field of the incident light, E_x, is wholly converted into the vertical field, E_z, necessary for ISBT. Consequently, the QW exhibits strong absorption (Supplementary Fig. 3b). Supplementary Fig. 3c suggests that the power converges at the QW. However, magnification of the power flow near the QW (Supplementary Fig. 3d) shows that the power that enters through the side faces splits into two flows, one aiming at the QW while the other at the inner Au walls. The spatial distribution of absorption was also calculated (Supplementary Fig. 3e). The majority of incident power is absorbed by Au and the contact layers; the share of the QW is limited to 25%. The employment of SWAs and tuning

of the resonance to the QW absorption peak are essential for realizing the maximum performance of the QWIP. However, the high g due to the choice of N_{qw} = 1 was necessary to achieve a conversion efficiency as high as 61%.

Supplementary Note 3. Propagation properties of plasmonic wires

An MIM waveguide of width L = 1.08 μm (Fig. 4e) exhibits a similar but slightly smaller wave vector (effective index: 3.0) than the infinitely wide waveguide[4,5] due to the edge effect. The electromagnetic fields in Supplementary Fig. 4a exhibit uniform E_z and H_y in the central region of the semiconductor layer, which are nearly equivalent to the lowest-order TM mode (E_x, E_z, H_y) in an infinitely wide MIM waveguide. However, other components are remarkable near the edges, thus, this mode is referred to as a TM-like mode.

In Fig. 4f, results necessary for various comparisons of k_{wire} are presented. Here, the physical length, D, of the wire per period (length in the x direction: $P-L$), $P-L+2S$ and $P-L+4S$ for Z- and S-shaped wires, respectively, is of importance. First, k_{wire} for the same folding geometry but with different dimensions, an S-shaped wire with S = 0.38 μm (D = 2.44 μm) and that with S = 0.225 μm (D = 1.82 μm), are compared. At a fixed photon energy (wavelength), the wire with S = 0.38 μm having a larger D exhibits a larger k_{wire}; thus, k_{wire} is larger for a wire with a longer physical length, which is natural. Next, a Z-shaped wire with S

= 0.45 μm and an S-shaped one with S = 0.225 μm have the same physical length (D = 1.82 μm), but exhibit different dispersions. Therefore, k_{wire} also depends on the folding shape. Finally, the Z-shaped wire with S = 0.45 μm and the S-shaped one with S = 0.38 μm support resonance at the same wavelength (Fig. 1) when combined with antennas to form SWAs, but have different dispersion properties. This straightforwardly suggests that the resonance of SWAs cannot be discussed with the simple combination of k_{antenna} and k_{wire}, which necessitates a more carel discussion of k_{1D}.

The electromagnetic modes of the wires of width W = 0.1 μm in Supplementary Fig. 4b−d are very different from the infinitely wide MIM waveguide, in which both the electric and magnetic fields are confined between the upper and lower Au layers. In a straight wire, the electric fields widely spread from the upper Au layer, and the upper layer is surrounded by magnetic fields. Therefore, the upper Au layer can be said to function as a single-layer plasmonic waveguide. Even if the wire is folded, the modes are nearly equivalent to a straight wire. Any deviation of the electromagnetic fields from a straight wire can only be observed in the cross-section B at the winding parts in the S-shaped wires.

Supplementary Fig. 5 shows the rotation of phase with respect to the physical length measured along the wires for various wire geometries. The slope represents the local phase velocity. The straight wire has the highest velocity; the length required for a certain phase

delay is minimum. The Z-shaped wire requires a slightly longer physical length for the same phase delay. The S-shaped wires need much longer length depending on their *S* values. In particular, the phase velocity shows a large deviation around the winding parts in the S-shaped wires, which corresponds to the deviated electromagnetic fields shown in Supplementary Fig. 4d (cross-section B). As a result of the accumulation of such phase evolution, the dependence of phase delays after the travel of $P-L$ in the x direction on the wire geometries is generated.

The numerically obtained propagation length expressed by the imaginary part of the wave vectors for the TM-like mode[5] is 8.5 μm for k_{antenna}, and 3–11 μm for k_{wire}. The propagation lengths of the wires are sufficient to form a coherence among the next antennas separated by a distance $P-L$ = 0.92 μm. In contrast, both of the antennas and wires do not support the propagation of transverse-electric-like modes (major components: E_y, H_x, and H_z in the coordinate system of Supplementary Fig. 4a).

The interpretation of these properties based on equivalent circuit presentation would be interesting, but it is out of the scope of this paper.

Supplementary Note 4. Dispersion properties of arrayed antennas

The array of isolated antennas does not exhibit diffraction because of the sufficiently small period ($P < \lambda/2$), and there is no propagation between the antennas. Therefore, the condition for the individual antennas given by equation (1) also holds for the resonance of the arrayed isolated antennas. The small energy discrepancy in Fig. 4a between the rigorous calculation (color map) and equation (1) (white broken line) is because the reflection phase of the gap mode at the cavity edge is not exactly π.

Equation (2) is based on the assumption that, in a combined system with a period P made of antennas of length L and a wire of length of $P-L$ in the x direction, the in-plane propagation can be effectively described with a simple combination of properties of the individual building blocks, k_{antenna} and k_{wire}. Except for Fig. 4b on straight wires, such a simple model reproduces both the resonance wavelengths and dispersion curves of the rigorous results fairly well.

The 1D array made of antennas and straight wires has blurred bandgaps due to the absorption loss, and k_{1D} exhibits vertical tails (near $k_x = 0$ in Fig. 4b and around BZ edges in Fig. 4g), which apparently suggests group velocities exceeding the light velocity. However, these regions do not contribute to the energy transport due to the large imaginary part of k_{1D}. Hence, this situation is physically allowed.

Figure 4c shows a remarkable horizontal mode denoted by the black arrow. However, this important feature is not explained in the main text at all. The origin of this mode can be discussed based on Supplementary Movie 1, which presents the distribution of E_z of the four SWAs shown in Fig. 1 for various incidence angles. In the Z-shaped SWAs with S = 0.45 μm, strong resonance is maintained in the square cavities throughout a wide incidence angle. Supplementary Movie 1 suggests that this resonance is excited by the mutual coupling in the y direction. Because the model in the main text only considered coupling in the x direction, this horizontal mode could not be described. In the Z-shaped SWAs, this resonance can be excited as the wires are connected with the antennas at asymmetric positions. In the S-shaped SWAs, however, the wires are attached at the centers of the edges; therefore, this resonance cannot be efficiently excited. Further details are not discussed in this paper.

Because the proposed SWA structures with $S \neq 0$ have no mirror symmetry, they must have chirality. However, this feature is also not discussed in this paper.

Supplementary Note 5. Properties of intermediate-shaped wires

In addition to the Z- and S-shaped wires, the intermediate-shaped wires were investigated. Results corresponding to Figs. 1 and 4, and Supplementary Fig. 5 are displayed in Supplementary Fig. 6. The physical length, D, of the intermediate-shaped wire per period

(length in the x direction: $P-L$) is given as $P-L+3S$. All the properties of the intermediate-shaped wires (except for the angle dependence, explained in Supplementary Note 1) were between those of the Z- and S-shaped wires. A scanning electron micrograph is also shown in Supplementary Fig. 1b. The responsivity and incidence-angle dependence are shown in Supplementary Fig. 1f and i.

Supplementary Note 6. Interpretation of enhancement factor to as high as 800

In the following two sections, other specific discussions related to the enhancement factor and strong coupling are presented to deepen the understanding of the obtained results.

The conversion efficiencies of the Brewster-angle and antenna-enhanced detectors were 0.076% and 61.0%, respectively, which yielded an enhancement factor of 803. This enhancement can be interpreted as follows.

According to Fresnel's analysis, the vertical electric field intensity $|E_z|^2$ at the QW in the Brewster-angle detector is approximately 0.02 (normalized by the incident field) for p polarization. This small value is due to the shallow incidence angle and high index of the semiconductor layer. On the other hand, the numerical simulation shows that the average field enhancement (averaged over the area of $P \times P$) at the QW layer incorporated in the SWA is typically $|E_z|^2 \sim 10$. Thus, an enhancement of 500 is achieved. This is the result of rotation and

squeezing of the incident field into the thin space between the metal layers. The Brewster-angle detector has no sensitivity to *s* polarization. This polarization dependence is completely removed by the SWAs, which adds an enhancement factor of 2. All these effects lead to a total enhancement of 1,000, which is nearly consistent with the observation. If the difference in g due to the peak V_b difference between the Brewster-angle and antenna-enhanced detectors (3.0 vs 2.3) were factored in, an enhancement of 770 is achieved, which is in excellent agreement with the experiment.

In the present responsivity, the photocurrent produced by the wire parts are included. According to the numerical simulation, the contribution of the wires is typically 10%.

Supplementary Note 7. Strong coupling

Todorov et al. have observed strong coupling (peak splitting) in similar systems even at room temperature[6–8]. In contrast, no splitting was observed in the absorption and responsivity spectra in this study. The proposed QWIP employs a bound-to-continuum scheme, thus, the assumption of the two-level system in the conventional cavity quantum electrodynamics (CQED) does not hold. Consequently, the absorption spectra of the proposed QW does not exhibit a single Lorentzian peak. Instead, they exhibit asymmetric shapes near the band edge. If the asymmetric spectra is regarded as a sum of the Lorentzian peaks, as we did in the fitting

of the dielectric function in the QW, the dominant contribution in the ISBT can be separated. Under these assumptions, the CQED parameters[9] are estimated as follows: coupling strength g_c = 4.1 meV[6], photon decay rate κ = 9.5 meV, and ISBT rate γ = 18.7 meV. Because $g_c^2 < (\kappa-\gamma)^2/4$, we are in a weak coupling regime. We adopted a single thin QW, and, therefore, the total electron density, thus g_c, is too small to observe the Rabi splitting.

Supplementary Note 8. Comparison with similar electromagnetic structures

Structures very similar to the SWAs presented here have been investigated in the gigahertz range[10,11] as the electromagnetic bandgap (EBG) structures[12], but for opposite functions of the SWAs in this paper. EBG structures provide bandgaps at the first BZ edge to minimize electrical noise; there are no electromagnetic modes inside and no interaction with external fields. In contrast, the SWAs in this paper support resonant modes in the antennas and offer enhanced interaction with external fields at the second BZ edge to maximize the sensitivity.

The structures proposed in this study could instead be viewed as two-dimensional (2D) leaky-wave antennas. 1D arrays of patch antennas simply connected by straight wires have long been known as typical leaky-wave antennas[13], and have been used for thermal emission[14]

and infrared detection[1,2]. However, SWAs put greater emphasis on the geometry of wires for highly engineered 2D networks of antennas.

The positive use of conducting wires for resonance control has recently attracted attention in the terahertz region, too[15,16].

Supplementary Note 9. Phase-gradient SWAs for wavefront-selective photodetectors

We numerically show that a photodetector with selective sensitivity for an incident cylindrical wave with a specific curvature can be realized by the gradual change in the wire geometry. The discussion is based on the SWA shown in Fig. 1d with S-shaped wires (L = 1.08 μm and P = 2.0 μm). The wires in the y direction are assumed to be straight for simplicity. In the x direction, nineteen antennas with the same dimension (electrical area) are connected with S-shaped wires with position-dependent S values. The S value of the wire interconnecting the i-th and (i+1)-th antennas from the center antenna (i = 0) is defined as S_i. The outermost wires are connected to perfect matching layers. The structure is shown in Supplementary Fig. 8a.

A y-polarized magnetic line source is placed at distance Z_s in the z direction above the center antenna. The incident electric field at the center antenna is x-polarized, as in Fig. 1.

Based on the dispersion relation of the antennas connected with S-shaped wires in Fig. 4g, the S_i values (in μm) were determined so that each antenna is synchronously resonated to the obliquely incident light corresponding to each position for a light source at λ = 6 μm and Z_s = 10 μm; S_0 = 0.25, S_1 = 0.30, S_2 = 0.35, S_3 = 0.38, S_4 = 0.41, S_5 = 0.43, S_6 = 0.44, S_7 = 0.45, S_8 = 0.46, and S_9 = 0.47.

Numerical simulation revealed that the expected wavefront dependence is obtained at λ = 6.1 μm. The Z_s dependence of the absorption of the SWA structure is shown in Supplementary Fig. 8b. The absorption exhibits the maximum of 0.74 at Z_s = 10 μm. If a QWIP layer with a sensitivity peak at λ = 6.1 μm is incorporated into this SWA, an infrared photodetector with a selectively high responsivity for a cylindrical wave with a curvature radius of Z_s = 10 μm is achieved. The results discussed in Fig. 1 can be interpreted as the design solutions for normally incident planar wavefronts ($Z_s \rightarrow \infty$).

Thus, photodetectors in which the function of a specific optics is integrated are realized by optimizing the position-dependent wire geometry. If both the wires and antennas are gradually changed, further complicated functions could be designed. Previously, such wavefront-sensitive functions have been realized by phase-gradient metasurfaces. However, phase-gradient SWAs offer electrically connected antennas; therefore, they are promising for optoelectronic devices sandwiching QWIP layers or other functional layers. Moreover, due to

their higher design freedom, SWAs would also find applications as new types of phase-gradient metasurfaces without electronic functions.

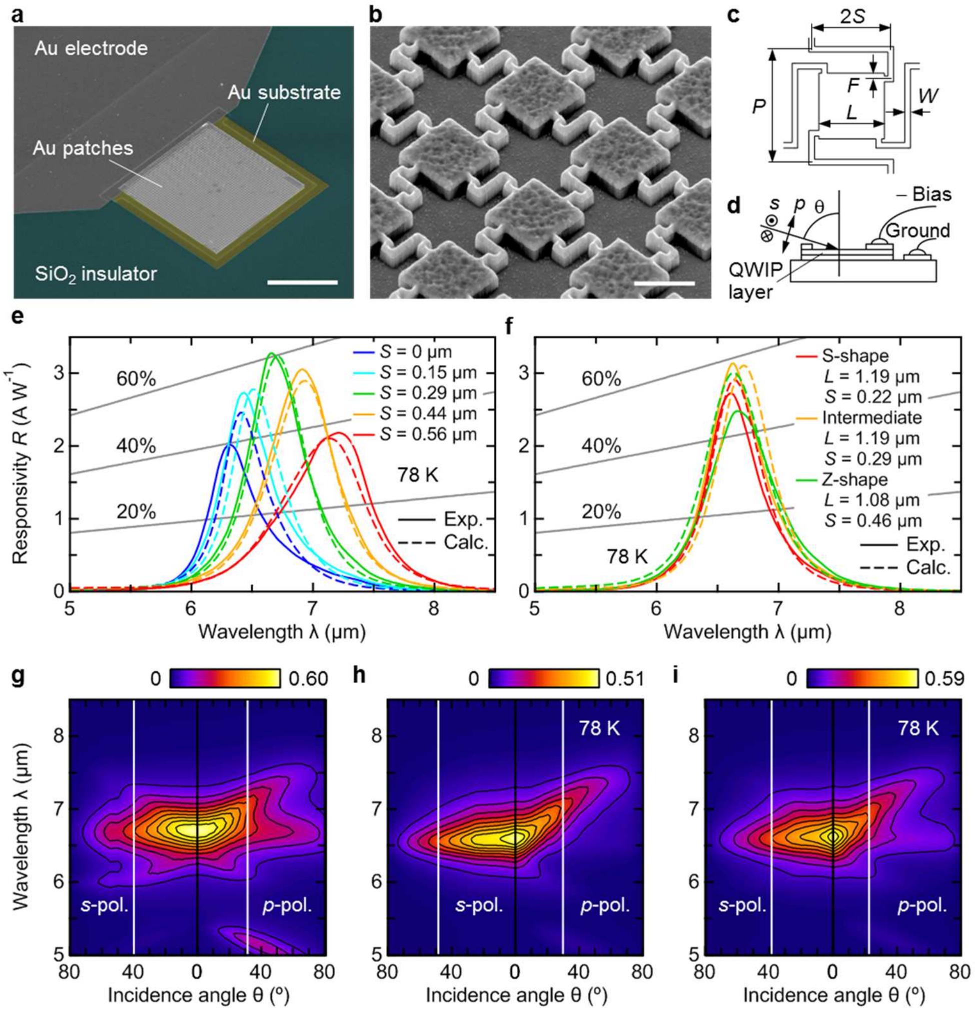

Supplementary Fig. 1. Additional results on the fabricated antenna-enhanced detectors.

a, Scanning electron micrograph showing the global image of a detector. Bar: 50 µm. **b**, SWAs with intermediate-shaped wires (*L* = 1.19 µm, *S* = 0.29 µm). Bar: 1 µm. **c**, Definition of Z-shaped SWAs with a large *S* value. **d**, Incidence for a Brewster-angle detector. **e**, Experimental and numerical responsivity spectra for Z-shaped SWAs with various *S* values (Fig. 3d, bottom), and **f**, other SWAs (S- and intermediate-shaped SWAs with *L* = 1.19 µm,

and Z-shaped ones with L = 1.08 μm). Equiefficiency (η_{conv}) lines are also shown. **g**, Numerical θ dependence of η_{conv} for *p*- (right) and *s*-polarizations (left) corresponding to the experiment in Fig. 3f (Z-shaped, L = 1.19 μm, S = 0.29 μm). **h** and **i**, Experimental θ dependence of η_{conv} for S- (L = 1.19 μm, S = 0.22 μm) and intermediate-shaped SWAs (L = 1.19 μm, S = 0.29 μm), respectively.

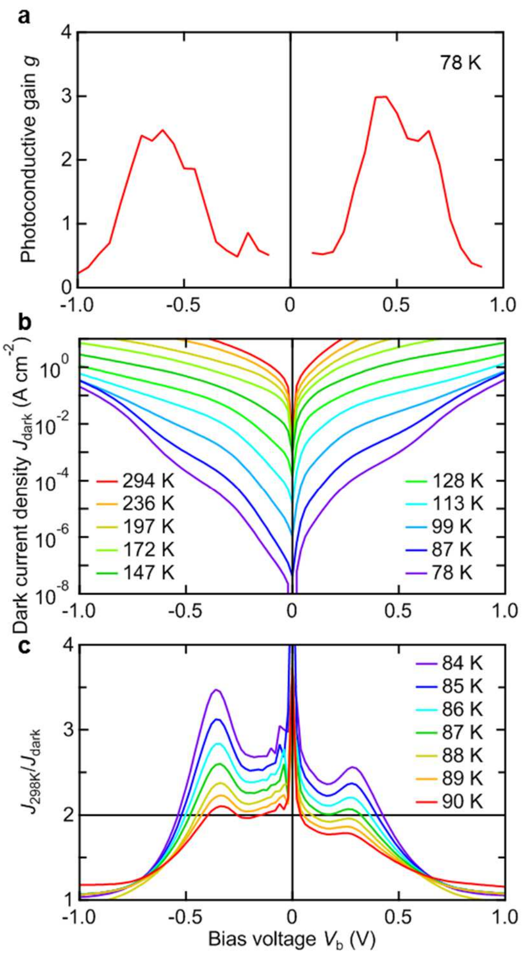

Supplementary Fig. 2. Bias dependence of currents in the Z-shaped SWAs with *S* = 0.29 μm, which exhibited the maximum responsivity. a, Photoconductive gain *g* determined from the dark-current noise spectra. **b**, Temperature dependence of dark current density, J_{dark}. **c**, Ratio of the background, J_{298K}, to the dark current, J_{dark}, at various temperatures.

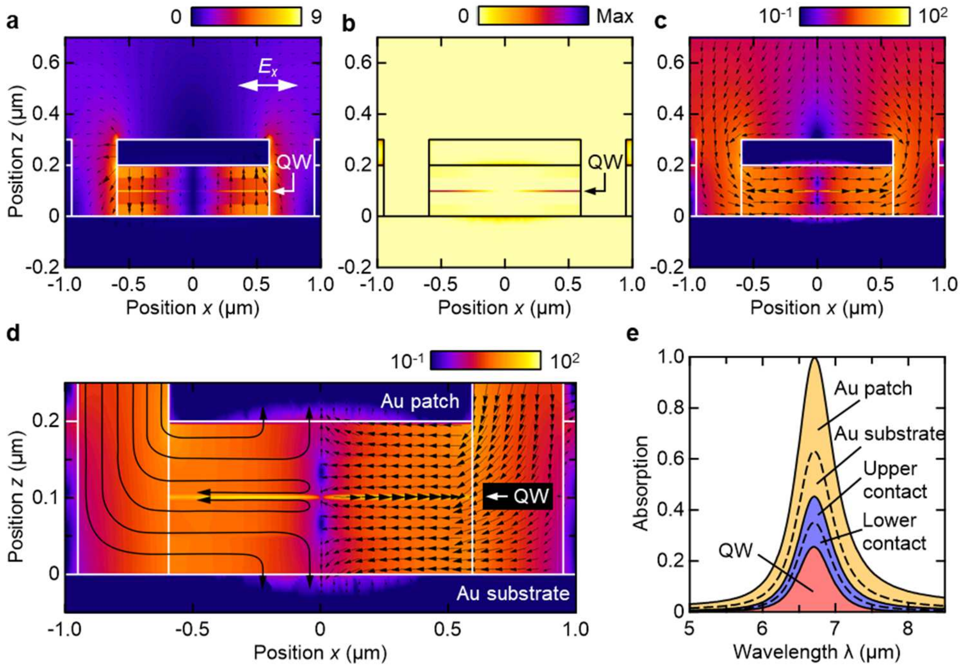

Supplementary Fig. 3. Numerically obtained electromagnetic response in the Z-shaped SWAs with S = 0.29 μm, which exhibited the maximum responsivity. The fields are for x-polarization, θ = 0°, and λ = 6.7 μm (the peak wavelength in the numerical absorption spectrum). **a**, Electric field amplitude E_z (color) and vector (E_x, E_z) at the moment of the maximum field at the QW, normalized by the incident field. **b**, Absorbed power density normalized by the maximum. **c**, Time-averaged power flow normalized by the incident power. The intensity shows the magnitude of the Poynting vector, log|(S_x, S_y, S_z)|. The vector shows the direction of the Poynting vector, whose magnitude is proportional to log(|(S_x, S_z)|/0.1)[17]. **d**, Detailed power flow near the QW. Schematic flow lines are illustrated on the left half for reference. The Poynting vector finally disappears in the Au layer or the QW. **e**, Share of the absorbed power of each layer.

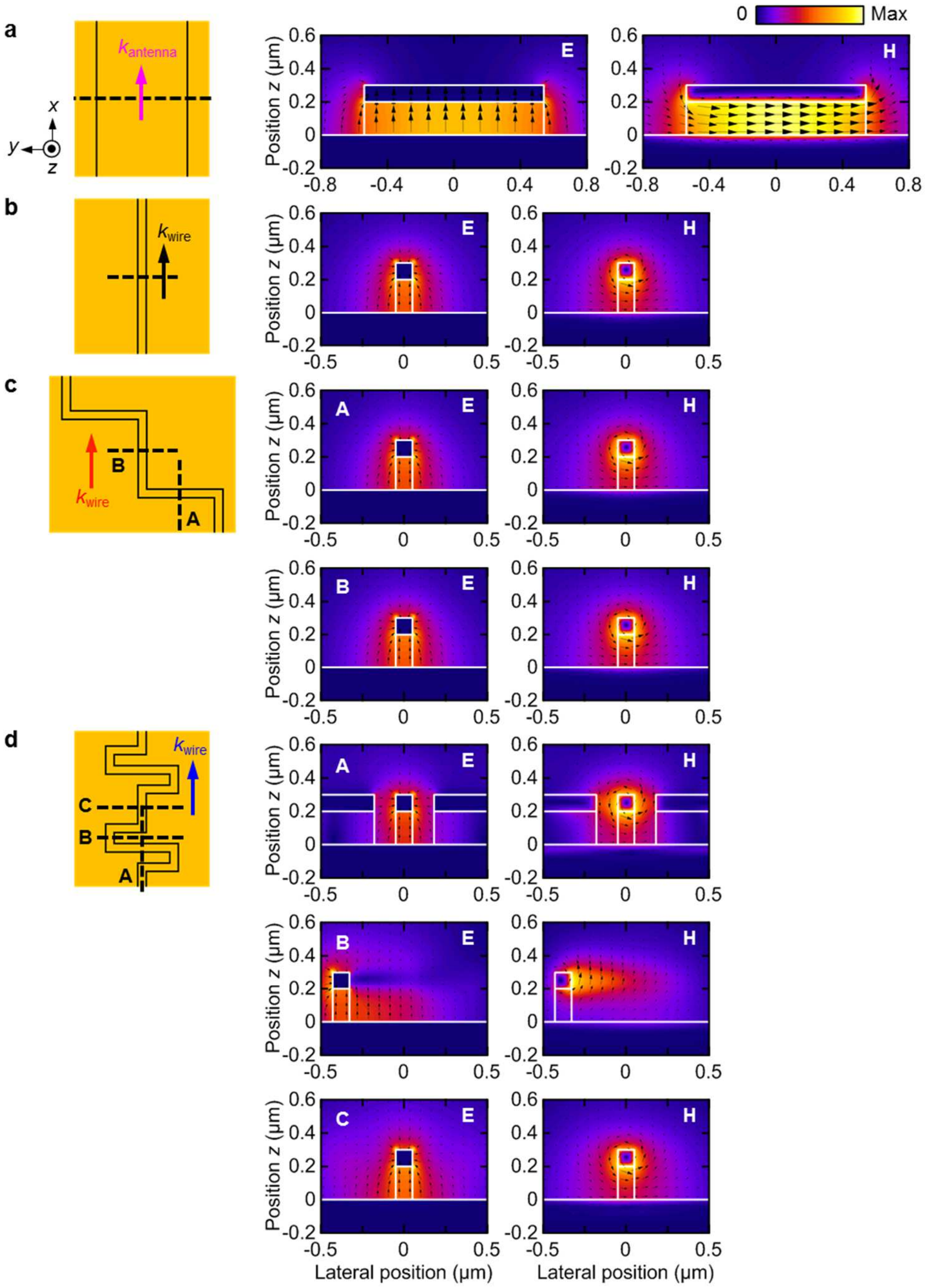

Supplementary Fig. 4. Electromagnetic modes in the MIM structures used for the antennas and wires. The wave vectors and cross-sections considered (broken lines) are defined in the schematics (left). The distribution of electric (center) and magnetic fields (right) at the moment that the E_z just below the upper Au exhibits the maximum amplitude.

The fields are normalized by the maximum value in each panel. The color indicates the magnitude of the electric (magnetic) field. The vectors show the in-plane components in each cross-section. **a**, MIM waveguide constituting the antennas (width: L = 1.08 μm). **b**, MIM waveguide (width: W = 0.1 μm) used as a straight wire. **c**, Z-shaped wire with S = 0.45 μm. The fields at two cross-sections are identical. **d**, S-shaped wire with S = 0.38 μm. The fields at the edge of the winding part (cross-section B) are different from those at other positions.

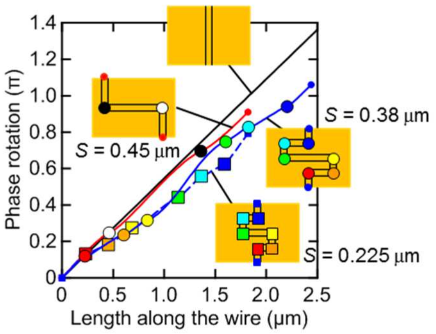

Supplementary Fig. 5. Phase evolution along the folded wires for various wires. The results for the same wires as in Fig. 4f are shown: straight, Z-shaped with S = 0.45 μm, S-shaped with S = 0.38 μm, and S-shaped with S = 0.225 μm. The structures corresponding to each curve are shown schematically. Colored marks on the curves correspond to the corners in the folded wires denoted by the same marks.

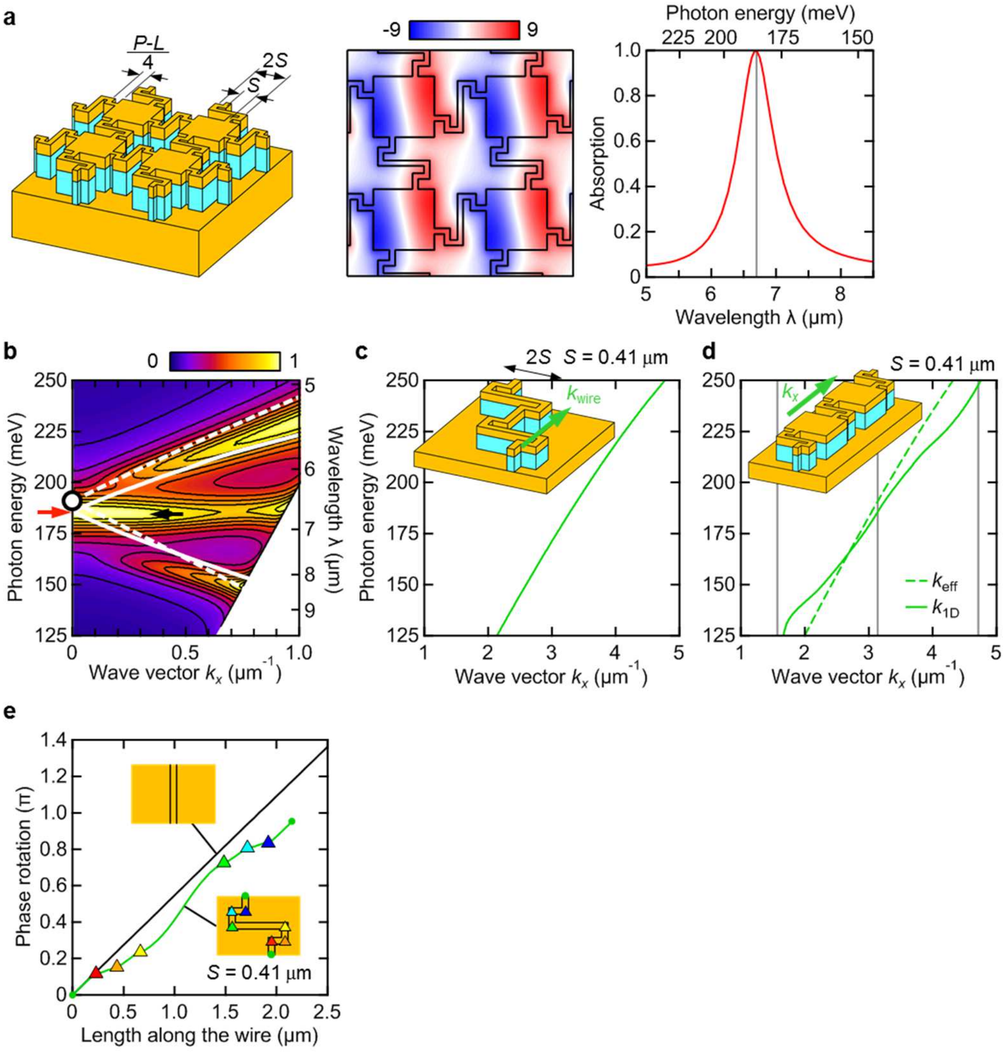

Supplementary Fig. 6. Properties of SWAs with folded wires with an intermediate shape. L = 1.08 μm, P = 2.0 μm, T = 200 nm, W = 100 nm, and S = 0.41 μm. **a**, Structure (left), E_z distribution at λ = 6.7 μm (center), and absorption spectra (right). **b**, Wave-vector dependence of absorption spectra for *p*-polarization incidence. **c**, Dispersion relations (real part) of TM-like propagation modes of infinitely repeated folded wires, and **d**, 1D periodic

system made of antennas and wires (solid curves). The broken lines are the dispersion relations of k_{eff} based on equation (2). The vertical gray lines denote the BZ edges. The dispersions of wired antennas represented by solid and broken lines in **d** are also displayed in **b**. The horizontal mode denoted by a black arrow in **b** cannot be discussed with the 1D periodic model. **e**, Phase evolution along the folded wires (see Supplementary Fig. 5).

Types		Yagi-Uda antennas	Phase-gradient metasurfaces	Global antenna mutual coupling	Synchronously wired antennas
Individual resonators	Shape	Unequal[a]	Unequal	Equal	Equal[b]
	Size	≈ λ/2	≪ λ	≪ λ	≪ λ
Arrange-ment	Period	≪ λ	≪ λ	≳ λ	≪ λ
	Coupling	Near field	No	Far field	Guided wave
Typical structures		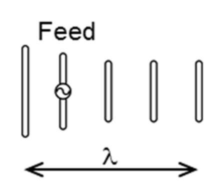	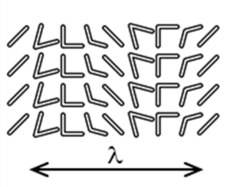	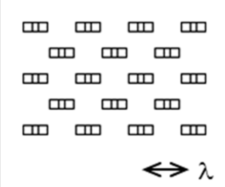	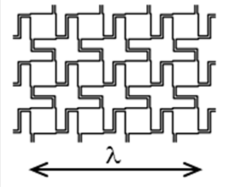

Supplementary Fig. 7. Comparison of four types of arrayed subwavelength resonators.

λ: wavelength of the electromagnetic wave. ≪ λ typically means size ≲ λ/6 and period ≲ λ/3. Notes: a, Only the feed element is emitting or receiving (absorbing). b, Can be unequal.

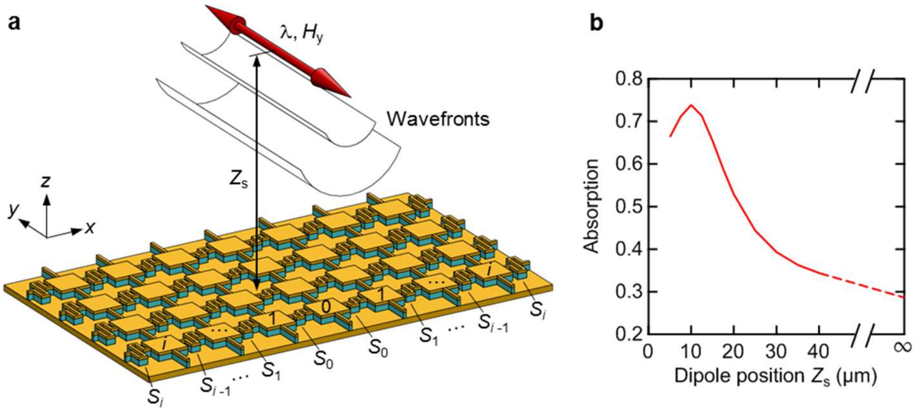

Supplementary Fig. 8. Phase-gradient SWAs for wavefront-selective photodetectors. a, Schematic illustration showing the patch antennas interconnected with gradually changing S-shaped wires in the *x* direction (not to scale). *L* = 1.08 µm, *P* = 2.0 µm, *T* = 200 nm, and *W* = 100 nm. The *S* values are *S* = 0.25, 0.30, 0.35, 0.38, 0.41, 0.43, 0.44, 0.45, 0.46, and 0.47 µm from the center to the edges. **b**, Dipole position dependence (curvature radius dependence) of the absorption of the phase-gradient SWA for a *y*-polarized linear magnetic dipole radiating λ = 6.1 µm at *z* = Z_s.

Supplementary References

1. Chen, Y. N. et al. Antenna-coupled microcavities for enhanced infrared photo-detection. *Appl. Phys. Lett.* **104**, 031113 (2014).
2. Palaferri, D. et al. Room-temperature nine-μm-wavelength photodetectors and GHz-frequency heterodyne receivers. *Nature* **556**, 85−88 (2018).
3. Schneider, H. & Liu, H. C. *Quantum Well Infrared Photodetectors: Physics and Applications* (Springer, Berlin, 2007).
4. Dionne, J. A., Sweatlock, L. A., Atwater, H. A. & Polman, A. Plasmon slot waveguides: Towards chip-scale propagation with subwavelength-scale localization. *Phys. Rev. B* **73**, 035407 (2006).
5. Kurokawa, Y. & Miyazaki, H. T. Metal-insulator-metal plasmon nanocavities: Analysis of optical properties. *Phys. Rev. B* **75**, 035411 (2007).
6. Todorov, Y. et al. Strong light-matter coupling in subwavelength metal-dielectric microcavities at terahertz frequencies. *Phys. Rev. Lett.* **102**, 186402 (2009).
7. Todorov, Y. et al. Ultrastrong light-matter coupling regime with polariton dots. *Phys. Rev. Lett.* **105**, 196402 (2010).
8. Jouy, P. et al. Transition from strong to ultrastrong coupling regime in mid-infrared metal-dielectric-metal cavities. *Appl. Phys. Lett.* **98**, 231114 (2011).

9. Agarwal, G. S. *Quantum Optics* (Cambridge Univ. Press, Cambridge, 2013).

10. Wu, T.-L. et al. A novel power plane with super-wideband elimination of ground bounce noise on high speed circuits. *IEEE Microw. Wireless Compon. Lett.* **15**, 174−176 (2005).

11. Qin, J. & Ramahi, O. M. Ultra-wideband mitigation of simultaneous switching noise using novel planar electromagnetic bandgap structures. *IEEE Microw. Wireless Compon. Lett.* **16**, 487−489 (2006).

12. Abhari, R. & Eleftheriades, G. V. Metallo-dielectric electromagnetic bandgap structures for suppression and isolation of the parallel-plate noise in high-speed circuits. *IEEE Trans. Microw. Theory Tech.* **51**, 1629−1639 (2003).

13. Derneryd, A. G. Linearly polarized microstrip antennas. *IEEE Trans. Antennas Propag.* **24**, 846−851 (1976).

14. Kinzel, E. C. et al. Directional thermal emission from a leaky-wave frequency-selective surface. *J. Nanophoto.* **9**, 093040 (2015).

15. Walther, C. et al. Microcavity laser oscillating in a circuit-based resonator. *Science* **327**, 1495−1497 (2010).

16. Paulillo, B. et al. Ultrafast terahertz detectors based on three-dimensional meta-atoms. *Optica* **4**, 1451−1456 (2017).

17. Miyazaki, H. T. & Kurokawa, Y. How can a resonant nanogap enhance optical fields by many orders of magnitude? *IEEE J. Sel. Top. Quantum Electron.* **14**, 1565−1576 (2008).
