# Supplementary material for: Synchronously wired infrared antennas for resonant single-quantum-well photodetection up to room temperature
Source: Nat Commun. 2020 Jan 28;11:565. doi: 10.1038/s41467-020-14426-6 (PMC6987185; doi:10.1038/s41467-020-14426-6)
Supplement: Supplementary file 3 — Description of Additional Supplementary Files [file 41467_2020_14426_MOESM3_ESM.pdf]

Title: Supplementary Movie 1.

Description: Electric field Ez for the representative arrayed patch antennas showing the difference in resonance due to the wires. a, Array of isolated antennas. b, Antennas connected with straight, c, Z-shaped (S = 0.45 µm), and d, S-shaped (S = 0.38 µm) wires. The common parameters are L = 1.08 µm, P = 2.0 µm, T = 200 nm, and W = 100 nm. The incidence angle is varied from θ = 0° to 80°. The incident light is p-polarized. The backward wave propagation due to negative dispersion can be seen in b. Remarkable coupling in the y direction responsible for the horizontal mode denoted by the black arrow in Fig. 4c can be observed in c.
